# Clinical, Serological and Immunological Characteristics in Greek Patients with Psoriatic Arthritis: The Role of IL-17, IL-23, and Sclerostin

**DOI:** 10.31138/mjr.31.2.235

**Published:** 2020-06-10

**Authors:** Aliki I. Venetsanopoulou, Theodora E. Markatseli, Michail P. Migkos, Athanasios Georgiadis, Foivos S. Kanellos, Alexandros A. Drosos, Paraskevi V. Voulgari

**Affiliations:** Rheumatology Clinic, Department of Internal Medicine, Medical School, University of Ioannina, Ioannina, Greece

**Keywords:** Psoriatic arthritis, interleukin 17, interleukin 23, sclerostin

## Abstract

Psoriatic arthritis (PsA) is an inflammatory form of arthritis that belongs to the family of spondyloarthritis (SpA) and is related to skin psoriasis. The incidence and prevalence of the disease vary considerably between countries. PsA is classified into axial PsA and peripheral PsA, with a wide range of other extra-articular manifestations. Although the aetiology of the disease is unknown, genetic, environmental, and immunologic factors appear to affect its appearance. In recent years, the role of the immune system in the pathogenesis of PsA has been increasingly investigated. Specific cytokines such as tumour necrosis factor (TNF), interleukin (IL-) 17 and IL-23, play an essential role affecting joint structures. This observation led to the emergence of tumour necrosis factor inhibitors (TNFi) that offer considerable therapeutic benefit to PsA patients. However, chronic inflammation causes bone loss, while new bone formation may also occur in both peripheral and axial skeleton. The molecular mechanisms underlying these processes have not yet been fully understood. So far, the role of the Wnt/β-catenin pathway and its inhibitors (Dickkopf and sclerostin) has been evaluated in ankylosing spondylitis (AS), but in PsA has not been studied sufficiently. The present study aims to investigate the epidemiological characteristics and clinical features (articular and extra-articular manifestations) as well as the treatment of PsA patients in the region of northwestern (NW) Greece. It also aims to evaluate the role of specific cytokines and sclerostin in patients with PsA, giving evidence to possible future biomarkers or even therapeutic targets for the disease.

## INTRODUCTION

Psoriatic arthritis (PsA) is a form of spondyloarthritis (SpA) that typically presents in people with skin psoriasis. Among psoriatic patients, PsA ranges from 4–30%. PsA affects men and women in a 1:1 ratio. The incidence and prevalence of the disease vary considerably between countries due to different diagnostic criteria, geographical and or population genetic characteristics.^1–5^

Clinical manifestations of PsA include peripheral arthritis (symmetric polyarthritis, asymmetric oligoarthritis, distal joint disease, arthritis mutilans) and axial involvement with sacroiliitis and spondylitis. Arthritis presents after the onset of psoriatic skin lesions in the majority of patients, but can also precede or coincide with psoriasis in 13–17%. Other clinical manifestations of PsA are dactylitis, enthesitis, tenosynovitis, eye inflammation (conjunctivitis, iritis), urinary tract involvement (urethritis) and, more rarely, pulmonary involvement (fibrosis), aortic regurgitation and amyloidosis.^5–7^ A number of other chronic inflammatory conditions occur more often in psoriatic patients such as inflammatory bowel disease (IBD) and celiac disease.^8^ While several diagnostic criteria have been proposed, there is no universal consensus. CASPAR (Classification criteria for psoriatic arthritis) criteria are an assessment tool for PsA with high specificity and sensitivity for the disease diagnosis.^7,9^ The laboratory findings are non-specific in PsA. Elevated acute-phase reactants (eg, C-reactive protein [CRP]) are frequently found in patients with active disease. Rheumatoid factor (RF), antinuclear antibodies, and antibodies to cyclic citrullinated peptides (anti-CCP) are usually negative.^10^

PsA is a disease of unknown cause. Genetic, immunological, and environmental factors contribute to its expression. The role of genetic factors is based on the high prevalence of the disease in families and monozygotic twins. Environmental factors that contribute to the pathogenesis of the disease include infections and trauma.^1–5,11,12^

Disease pathogenesis involves cytokines such as tumour necrosis factor-alpha (TNF-a) as well as interleukins (IL-) 17, 22, and 23. In particular, IL-23, which is mainly produced by dendritic cells, macrophages and keratinocytes, induces the expansion of the T helper (Th) 17 cells. An increased number of these cells have been found in the skin, synovial membrane, and synovial fluid of patients with PsA. Th17 cells produce IL-17 and promote synovial fibroblasts and macrophages to produce inflammatory cytokines, such as IL1-β, IL-6, and TNF-α, that ultimately cause bone destruction. In the enthesis, resident and infiltrating entheseal myeloid cells produce IL-23, which via IL23/IL-17, axis induce enthesitis.^13,14^ IL-23 also plays a Th17-independent role in bone homeostasis, as it promotes the expression of the Receptor Activator of Nuclear factor κB ligand (RANKL) in synovial fibroblasts and up-regulates the expression of the RANK in myeloid precursor cells, leading to osteoclast differentiation.^15^ Th17 cells are also the primary source of IL-22.^16^ IL-22 promotes the proliferation of human mesenchymal stem cells (MSCs) and induces differentiation to osteoblasts leading to new bone formation in entheses or around articular cartilages.^17^ Thus, the erosion caused by the inflammation «heals»: firstly, with the formation of fibrous tissue, and later with the endochondral bone formation. The bone formation is regulated by two systems: 1. Bone morphogenetic proteins that act under the influence of IL-1 and Th17 cell-secreted cytokines, and 2. Wnt/*β*-catenin pathway that promotes osteoblast formation and osteogenesis.^13,14,18^ Inhibitors of the Wnt pathway (Dkk1 [Dickkopf-1], sclerostin, and secreted frizzled-related proteins) have been studied in ankylosing spondylitis (AS) (found low or non-functional) and may contribute in osteogenesis.^19^ In PsA patients, Dkk-1 serum levels are found lower than in healthy controls,^20^ while a recent study by the same researchers showed that secukinumab (an anti-IL-17 antibody) produces a quick increase in Wnt signalling antagonists (sclerostin and Dkk-1) in PsA patients.^21^ However, this increase could be a direct consequence of the inhibition of the IL-17 pathway, or result of a counter-regulatory mechanism caused by the decrease in the action of IL-17 on osteoblast and osteocytes. Still, the role of sclerostin in combination with IL-17 and IL-23 cytokines in patients with PsA has not fully been studied.^15–19^

Treatment of PsA is often challenging and depends on the severity of the disease. There are many different treatment options available for PsA patients: non-steroidal anti-inflammatory drugs, corticosteroids, disease-modifying drugs such as methotrexate (MTX) or leflunomide, cyclosporine, sulfasalazine, and other agents such as apremilast, Janus kinase (JAK) inhibitors, and biological agents.^22^Apremilast is an orally-active small molecule that inhibits phosphodiesterase-4 (PDE4) and regulates inflammatory mediators. It is efficacious in the treatment of moderate-to-severe plaque psoriasis and has a favourable safety profile and efficacy across a broad range of the clinical features of PsA.^23^ JAK inhibitors are also a promising therapeutic option for PsA, and the selective JAK1/3 inhibitor tofacitinib is approved for the treatment of active PsA, where it is indicated in combination with MTX for patients who have had an inadequate response or who have been intolerant to prior therapy with a DMARD.^24^ Finally, biologic agents include TNFα inhibitors, secukinumab (anti-IL-17), ustekinumab (targets IL-12 / IL-23), while other monoclonal antibodies that selectively neutralize IL17A and IL17F or IL 23 are under investigation and some of them show promising preliminary results.^25^

## AIMS OF THE STUDY

The purpose of this study is to investigate, 1. the epidemiological, clinical, serological, and immunological characteristics of PsA in NW Greece, and 2. the serum levels of sclerostin and cytokines (IL-17 and IL-23) in PsA patients. A further correlation of these parameters with the patients’ clinical, laboratory characteristics and imaging findings will be examined, in relation to other factors that may affect their levels, such as bone mineral density (BMD) and anti-osteoporosis treatment. The results will possibly indicate new disease biomarkers and may have an impact on PsA treatment.

## METHODS

The study will include patients from NW Greece over the age of 18 who meet the CASPAR criteria and consent to participate in the study. Patients with positive RF, with other forms of SpA (AS, IBD, reactive arthritis) as well as patients with known pulmonary disease (tuberculosis, chronic obstructive pulmonary disease, sarcoidosis) and active hepatitis C or B will be excluded from the study. History and epidemiological data (including age, sex, duration of illness, and occupation) from all participants will be recorded. Patients will also undergo a full clinical examination with a record of articular (peripheral and axial) and extra-articular manifestations**.** X-rays from patients’ records will be evaluated for the presence of periostitis and joint damage, the involvement of the sacroiliac or spine joints and the existence of spurs at the entheses. Ultrasound examination of flexor and extensor tendons of fingers and toes will be performed in all patients with peripheral PsA by a Rheumatologist expert on musculoskeletal ultrasound and joint effusion, synovial proliferation, tenosynovitis and erosion of bone contour will be recorded. BMD using dual-energy X-ray absorptiometry (DXA) will be performed in the lumbar spine (L1–L4) and hip (femoral neck and total hip). Skin disease severity will be assessed using the PASI score (Psoriasis Area Severity Index), and peripheral joint activity by using the DAS-28 (28-joint Disease Activity) and the DAPSA (Disease Activity in Psoriatic Arthritis) score. The Health Assessment Questionnaire will be used to measure patients’ quality of life. The group of patients with axial involvement will be evaluated using the BASDAI (Bath Ankylosing Spondylitis Disease Activity Index) and the BASFI (Bath Ankylosing Spondylitis Functional Index). Finally, the patients’ treatment, since the disease diagnosis, including anti-osteoporotic drugs, will be recorded. All patients will undergo routine laboratory testing, hepatitis (B and C) serology, and immunological testing (RF, antinuclear antibodies, anti-CCP). An additional serum sample from all patients will be stored at −80° C, and the measurement of sclerostin, IL-17, and IL-23 (by ELISA method) will be made. The findings of these tests will be correlated to clinical and imaging findings and other serological and immunological parameters.

## STATISTICAL ANALYSIS

Microsoft Excel 2017 and Statistica v.12 will be used for the analysis. The data will be analysed using descriptive statistics. The continuous variables will be described by their mean and median values and their constant deviations and range. Categorical variables will be described as percentages of the total available records. Finally, multivariate analysis will be performed to identify the independent risk factors for each of the variables.

## STUDY APPROVAL

The study has been approved by the Ethics Committee of the University Hospital of Ioannina.
